# Disrupted Regional Homogeneity in Major Depressive Disorder With Gastrointestinal Symptoms at Rest

**DOI:** 10.3389/fpsyt.2021.636820

**Published:** 2021-05-26

**Authors:** Meiqi Yan, Jindong Chen, Feng Liu, Huabing Li, Renzhi Huang, Yanqing Tang, Jingping Zhao, Wenbin Guo

**Affiliations:** ^1^Department of Psychiatry, National Clinical Research Center for Mental Disorders, The Second Xiangya Hospital of Central South University, Changsha, China; ^2^Department of Radiology, Tianjin Medical University General Hospital, Tianjin, China; ^3^Department of Radiology, The Second Xiangya Hospital of Central South University, Changsha, China; ^4^Hunan Key Laboratory of Children's Psychological Development and Brain Cognitive Science, Changsha, China; ^5^Department of Psychiatry, The First Affiliated Hospital of China Medical University, Shenyang, China; ^6^Department of Psychiatry, The Third People's Hospital of Foshan, Foshan, China

**Keywords:** major depressive disorder, regional homogeneity, gastrointestinal symptoms, magnetic resonance imaging, resting state

## Abstract

**Background:** Gastrointestinal (GI) symptoms are prominent in patients with major depressive disorder (MDD). Previous studies have reported brain structural and functional changes in both MDD and digestive system diseases but it remains unclear whether MDD patients with GI symptoms have brain imaging changes.

**Methods:** We recruited 35 MDD patients with GI symptoms, 17 MDD patients without GI symptoms and 28 age-, gender-, and education-matched healthy controls. All participants were scanned by resting-state functional magnetic resonance imaging (fMRI). Imaging data were analyzed with regional homogeneity (ReHo).

**Results:** The GI group showed higher total HRSD-17 scores, anxiety/somatization, weight loss, and sleep disturbance scores compared to the non-GI group. We found increased ReHo in the right inferior parietal gyrus (IPL), bilateral supplementary motor area (SMA), bilateral cerebellum Crus II, left inferior frontal gyrus (IFG), and bilateral superior medial frontal cortex (SMFC) and decreased ReHo in the right posterior cingulate cortex (PCC), bilateral cuneus, and left middle occipital gyrus (MOG) in patients with GI symptoms relative to the HCs. The GI group showed higher ReHo values in the bilateral precuneus than the non-GI group.

**Conclusion:** MDD patients with GI symptoms showed a greater severity of symptoms than MDD patients without GI symptoms, particularly in terms of anxiety/somatization, weight loss, and sleep disturbances. Increased activity in the default-mode network might be associated with GI symptoms in MDD patients.

## Introduction

Major depressive disorder (MDD) is a common mental disorder globally, regardless of the level of income (1). The WHO predicts that MDD is set to become the world's largest cause of burden of disease by 2030 (2). Patients with MDD often have many somatic symptoms (like insomnia, pain, tachycardia, gastrointestinal symptoms, etc.), of which gastrointestinal (GI) symptoms (like gastralgia, gastric distention, nausea, vomiting, heartburn, acid reflux, constipation, diarrhea, etc.) are the most prominent symptoms. A previous study reported that elderly patients with MDD had a high proportion of anxiety (72.28%) and somatic symptoms (98.8%) (3). In an international study, half of a total of 1,146 patients with MDD reported multiple medically unexplained somatic symptoms and 11% of them denied any psychological symptoms of depression (4). Chronic diarrhea and constipation were significantly more common and prevalent in depressed individuals than non-depressed individuals (5). Gastrointestinal complaints such as diarrhea, abdominal pain, dyspepsia, constipation, or IBS occurred in 54% of subjects with depressive symptoms while it only occurred in 29% of subjects without depressive symptoms (6). A previous study indicated that somatic symptoms in MDD were associated with more severe clinical symptoms, lower remission rates (7), and a worse prognosis (8). However, due to the scarcity of mental health resources, coupled with the social stigma of mental disorders, patients with MDD accompanied with somatic symptoms tended to visit general hospitals for the first time or even repeatedly. However, it is difficult for a general physician to recognize mental illnesses like MDD, whereas a somatic chief complaint would increase the difficulty of identification (9), resulting in a long-term lack of correct diagnosis and effective treatment (10, 11). People generally call this condition “masked depression,” in which the depressive mood and cognitive symptoms of depression are hidden behind various somatic complaints or behavioral problems (12). Consequently, the social economic burden is increased because of the increased use of healthcare resources during episodes of MDD (13) and because of the additional medical costs caused by somatic symptoms (10). Thus, early and effective identifications of MDD with GI symptoms as their chief complaint are important for the prognosis of MDD. Furthermore, the mechanism of GI symptoms of MDD also needs to be revealed which may provide new ideas for therapeutic research.

Many digestive system diseases exhibit high rates of depression. A meta-analysis reviewed that the prevalence of depressive symptoms and depression in patients with irritable bowel syndrome (IBS) was estimated to be 28.8 and 23.3%, respectively, and they were three times more likely to suffer from anxiety or depression than healthy subjects (14). In a previous study, 44.4% of patients with inflammatory bowel disease (IBD) were reported to have anxiety or depression or both, leading to an increased use of medical resources (15). Additionally, the risk of depression 5 years after surgery in ulcerative colitis (UC) and Crohn's disease (CD) patients was 11% and 16%, respectively (16).

Inflammation is considered to be one of the pathogenesis of MDD (17, 18). In this process, the gut-brain axis (GBA) may play a critical role and is believed to be one possible critical mechanism of affective disorders (19). A previous study has reported that microbiota in the gastrointestinal tract (GI) can activate the neural pathways and the central nervous system (CNS) signaling systems, thereby affecting the related symptoms of MDD (20). However, it remains unclear whether MDD patients with GI symptoms have brain imaging changes. Many previous studies have reported that patients with MDD have brain structural (21–23) or functional (24–27) changes. Moreover, other studies have observed abnormal structural and functional brain MRI data in digestive system diseases, like irritable bowel syndrome (IBS) (28, 29), other functional bowel disorders (30), and inflammatory gastrointestinal diseases (31). A few scientists have tried to examine whether MDD patients with GI symptoms had brain imaging changes and a previous study reported that MDD patients with GI symptoms showed significantly lower gray matter volume (GMV) and regional homogeneity (ReHo) in the left middle frontal gyrus, precentral gyrus, right superior frontal gyrus, and the middle frontal gyrus, as well as higher ReHo in the left superior temporal gyrus, compared to MDD patients without GI symptoms (32).

Aberrantly increased amplitude of low-frequency fluctuation (ALFF) and functional connectivity (FC) were observed in the default-mode network (DMN) in patients with UC (33). Visceral sensory abnormalities are very common in patients with IBS (29). The DMN was reported to connect to gastric sensations (31), which is mainly comprised of the medial prefrontal cortex (MPFC), posterior cingulate cortex (PCC), precuneus, and lateral parietal cortex (34). Somatic symptom disorder may be associated with the altered processing of sensory discrimination of pain and other somatic symptoms (35). Further, a previous study suggested that brain regions involved in pain sensory processing shifted to those involved in subjective states of emotion and motivation in the majority of chronic pain diseases (36). Moreover, studies reported that chronic visceral pain might lead to functional reorganization in the DMN (37–39), thus, the DMN may exhibit certain changes in MDD patients with GI symptoms.

In this work, we performed a ReHo analysis to compare the differences between MDD with GI symptoms, MDD without GI symptoms and healthy controls. We hypothesized that: (1) increased ReHo in the DMN would be observed in MDD patients with GI symptoms; (2) increased ReHo might be correlated with clinical features of the patients.

## Methods

### Participants

A total of 52 patients aged between 18 to 55 years were recruited and divided into two groups based on the presence or absence of GI symptoms. The GI group consisted of all patients with at least one GI symptom (35 patients) and patients without GI symptoms were assigned to the non-GI group (17 patients). The GI symptoms mainly included medically unexplained gastralgia, gastric distention, nausea, vomiting, heartburn, acid reflux, constipation, diarrhea, etc. All patients were outpatients from the Second Xiangya Hospital of Central South University, China. The patients were diagnosed by two psychiatrists independently using the DSM-5 criteria for MDD. All patients included in this study met the following criteria: (1) first major depressive episode; (2) with the total scores of 17-item Hamilton Rating Scale for Depression (HRSD-17) (40) ≥17; (3) no history of antipsychotic therapy and electroconvulsive therapy (ECT); (4) no digestive diseases.

A total of 28 age-, gender-, and education- matched healthy controls were recruited from the community. They were excluded if they had a family history of mental disorders. They were also excluded if they had any history of neurological diseases, digestive diseases, substance abuse, or psychotic symptoms.

All participants were right-handed and Han Chinese. Exclusion criteria for all participants were as follows: (1) other psychiatric disorders meeting the DSM-5 diagnostic criteria; (2) any history of neurological illnesses, severe physical illnesses, and substance abuse; (3) pregnancy; (4) abnormal cerebral structure after initial MRI scan; (5) any contraindications for the MRI scan.

The 17-item HRSD was applied to evaluate the severity of MDD. It can be classified into the following five types of factors: (1) anxiety/somatization (six items containing psychic anxiety, somatic anxiety, gastrointestinal symptoms, hypochondriasis, insight, and general symptoms); (2) weight loss (one item); (3) cognitive disturbances (three items containing self-guilt, suicide, and agitation); (4) disability symptoms (four items, containing depression, work and interests, intellectual disability, and sexual symptoms); (5) sleep disturbances (three items, containing difficulty falling asleep, superficial sleep, and early awakening). Item 12 (GI symptoms) of the HRSD-17 was used to evaluate the severity of the GI symptoms; a score of 0 means no GI symptoms, scores of 1 and 2 mean having GI symptoms where a score of 1 indicates mild-to-moderate GI symptoms and a score of 2 indicates severe GI symptoms.

The study was approved by the Medical Research Ethics Committee of the Second Xiangya Hospital of Central South University, China. The study was conducted in accordance with the Helsinki Declaration. Each participant provided informed consent prior to enrollment.

### Image Acquisition

The resting-state MRI data were scanned by a 3.0 T Siemens scanner (Germany) in the Second Xiangya Hospital of Central South University, China. The echo planar imaging (EPI) sequence was applied to obtain the resting-state functional images using the following parameters: repetition time/echo time (TR/TE) 2,000/30 ms, 30 slices, 64^*^64 matrix, 90°flip angle, 24 cm field of view, 4 mm slice thickness, 0.4 mm gap, and 250 volumes lasting for 500 s.

### Data Preprocessing

Data preprocessing was conducted in MATLAB (MathWorks) by using Data Processing Assistant for Resting-State fMRI (DPARSF) (41). The instability of the initial MRI signals and the subject's acclimatization time may have affected the data results. To reduce the possible errors caused by these potential factors, the first 10 original images were discarded. The images were then corrected for slice-timing and head motion (maximum displacement in x, y, or z axis: 2 mm, maximum angular rotation: 2°). Next, the corrected images got spatial normalization to the MNI space with 3 × 3 × 3 mm^3^. After that, the fMRI data were filtered by temporal band-pass (0.01–0.08 Hz) and linearly detrended. Several spurious covariates, like signals from the center region of white matter (WM) and the region of interest (ROI) based on ventricular seeds, as well as the 24-head motion parameters obtained by the rigid body correction, were removed. The global signal was retained during the pre-processing of the resting-state FC data referring to a previous study (42).

### ReHo Analysis

We conducted the regional homogeneity (ReHo) analysis to study functional synchronization using an in-house software, REST (http://www.resting-fmri.sourceforge.net). Based on the assumption that a voxel and those of its neighbors were temporally similar, the Kendall's coefficient of concordance (KCC) was used to compare the similarities of the time series of one given voxel and its closest neighbor voxels in a voxel-wise analysis (43). The calculation formula of KCC has been expounded in a previous study (44). We obtained the individual ReHo map by calculating the KCC values of the time series of a given voxel with its nearest voxel (26 voxels) in a voxel-wise analysis. To reduce the impacts of individual variation in the KCC value, we divided the KCC of each voxel by the mean KCC of the whole brain to normalize the ReHo maps. Then the generated imaging data were spatially smoothed with a Gaussian kernel of 4 mm full-width, at half-maximum.

### Statistical Analyses

Group differences in age, years of education, HRSD-17 scores, and the five subscale scores of HRSD-17 across the three groups were compared by analysis of variance (ANOVA) in SPSS19.0 (LSD between two group comparisons). Gender distribution was described by performing a Chi-square test. We used a two-sample t-test to compare group differences of illness duration between the two patient groups. *P* < 0.05 was considered statistically significant.

The group differences were identified by performing ANCOVA in a voxel-by-voxel manner in individual whole-brain ReHo maps across the three groups, followed by *post-hoc t*-tests. Age, years of education, and framewise displacement were applied as covariates. The results were FDR (false discovery rate) corrected at *p* < 0.05.

### Correlation Analyses

We extracted average ReHo values from the brain regions with abnormal ReHo. The correlations between abnormal ReHo and HRSD-17 scores, the five subscale scores as well as the severity of the GI symptoms were assessed by Pearson's correlation analysis with a threshold of Benjamini-Hochberg corrected *p* < 0.05.

## Results

### Demographic Characteristics and Clinical Information

None of the participants were excluded due to excessive head movement. No group differences in age, years of education, and gender distribution were observed across the three groups, and illness durations did not significantly differ between the two patient groups. The HRSD-17 total and subscale scores (excepting weight loss) were all higher in two patient groups than healthy controls (HCs). The GI group showed higher weight loss scores than the non-GI group and HCs, whereas no significant difference in weight loss scores was found between the non-GI group and HCs. Furthermore, the GI group showed higher HRSD-17 total scores, anxiety/somatization, weight loss, and sleep disturbances scores than the non-GI group (Table 1).

**Table 1:** Demographic and clinical characteristics of the participants.

	**S1 (*n* = 35)**	**S0(*n* = 17)**	**HC (*n* = 28)**	***F‵t*or *χ^2^* value**	***Post hoc t*-tests or *p* values**
Age (years)	30.86 ± 6.84	30.29 ± 8.05	30.14 ± 5.00	0.102	0.903^a^
Gender (male/female)	13/22	6/11	14/14	1.377	0.502^b^
Handedness (Right/Left)	35/0	17/0	28/0		
Education (years)	14.51 ± 3.28	12.94 ±3.46	14.61 ±2.69	1.797	0.173^a^
Illness duration (months)	6.23 ± 4.63	6.94 ± 3.98		0.544	0.589^c^
HRSD-17 scores	22.69 ± 3.41	20.18 ± 2.67	0.89 ± 0.88	585.979	S1 > S0 > Nor
Anxiety/Somatization	7.31 ± 1.92	6.41 ± 1.66	0.39 ± 0.57	174.531	S1 > S0 > Nor
Weight loss	0.80 ± 0.83	0.06 ± 0.24	0	18.741	S1 > S0, Nor
Cognitive disturbances	3.71 ± 1.78	3.41 ± 1.50	0	64.213	S1, S0 > Nor
Retardation symptoms	6.40 ± 1.42	6.76 ± 1.56	0.18 ± 0.39	253.030	S1, S0 > Nor
Sleep disturbances	4.46 ± 1.42	3.53 ± 1.28	0.32 ± 0.55	103.570	S1 > S0 > Nor

*HRSD-17, 17-item Hamilton Rating Scale for Depression; S1, gastrointestinal (GI) symptoms group; S0, non-gastrointestinal (non-GI) symptoms group*.

a *The p-value was obtained by analyses of variance*.

b *The p-value was obtained by a Chi-square test*.

c *The p-value was obtained by two-sample t tests*.

### ReHo Differences Across Groups

**Figure 1:**
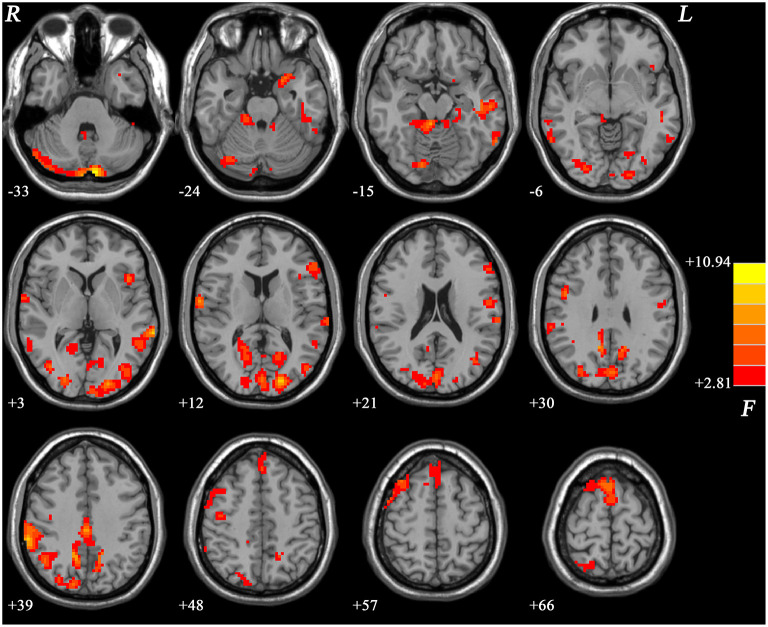
Brain regions showing differences in ReHo values across the three groups. The color bar indicates *F* values from ANCOVA (age, years of education, and framewise displacement as covariates). ReHo, regional homogeneity; ANCOVA, analysis of covariance. The results were FDR (false discovery rate) corrected at *p* < 0.05.

The differences of ReHo values showed significant differences mainly in the frontal, parietal, occipital, cerebellar, limbic, and cortical motor regions across the three groups (Figure 1).

#### MDD With GI Symptoms vs. MDD Without GI Symptoms

**Figure 2:**
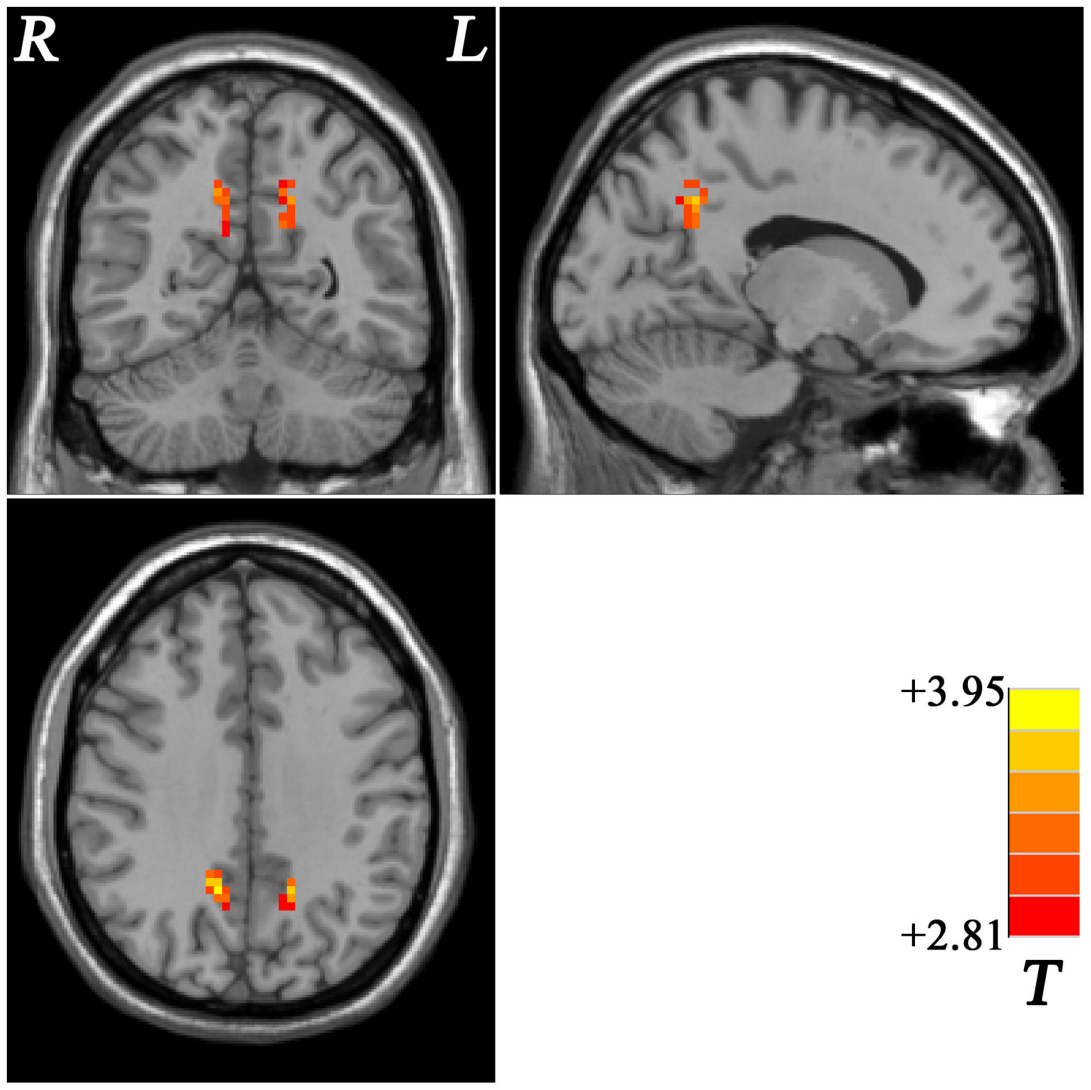
Statistical map depicts higher ReHo in GI group compared with non-GI group. The threshold was FDR (false discovery rate) corrected at *p* < 0.05. Red denotes higher ReHo in the GI group. Th color bar indicates *T* values from *post-hoc t*-tests. L, lest side; R, right side; GI, gastrointestinal, ReHo, regional homogeneity.

**Figure 3:**
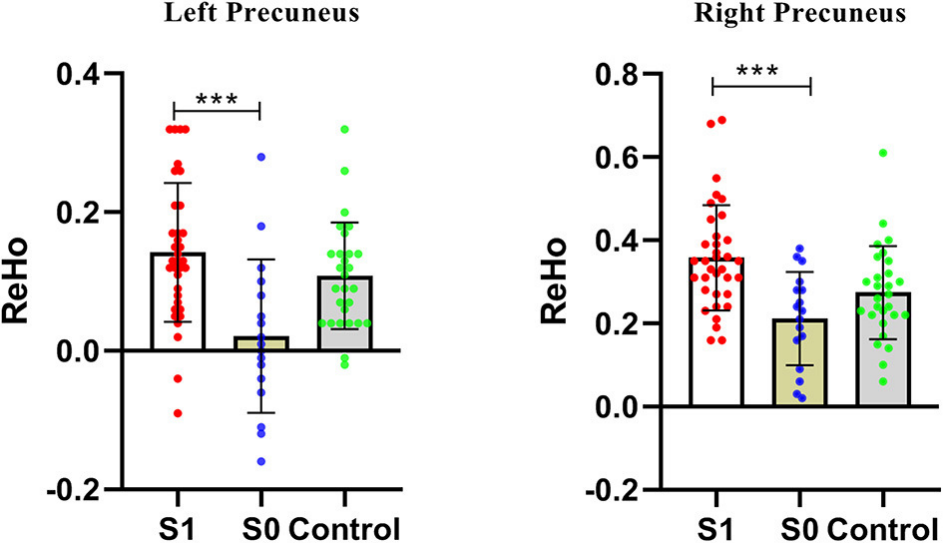
Comparison of ReHo of the bilateral precuneus across three groups. S1, gastrointestinal symptoms group; S0, non-gastrointestinal symptoms group; *** indicates *p* < 0.001.

Compared with the non-GI group, the GI group showed higher ReHo values in the bilateral precuneus (Figure 2, Table 2). No decreased ReHo in any brain regions was found in the GI group relative to the non-GI group. Since there was no significant difference in the bilateral precuneus between each patient group and the HCs, we examined the ReHo values of the bilateral precuneus in all three groups. As shown in Figure 3, the GI group showed higher ReHo in the left precuneus (*p* = 0.0001) and the right precuneus (*p* = 0.0002) compared to the non-GI group after correction.

**Table 2:** Significant ReHo differences across groups.

**Cluster location**	**Peak (MNI)**			**Number of voxels**	***T* value**
	**x**	**y**	**z**		
**S1 vs. S0**					
Left Precuneus	−15	−54	36	31	3.6427
Right Precuneus	12	−51	33	42	3.9460
**S1 vs. HC**					
Right Inferior Parietal Lobule	63	−39	39	43	3.4750
Bilateral Supplementary Motor Area	3	18	69	143	3.8175
Bilateral Cerebellum Crus II	−9	−90	−33	76	4.5214
Right Posterior Cingulate Cortex	18	−54	9	43	−3.2237
Bilateral Cuneus	0	−81	27	157	−4.0568
Left Middle Occipital Gyrus	−21	−93	6	96	−3.9454
**S0 vs. HC**					
Left Inferior Frontal Gyrus	−54	30	15	42	3.9354
Bilateral Superior Medial Frontal Cortex	0	39	51	66	3.8583

*MNI, Montreal Neurological Institute; ReHo, regional homogeneity*.

*S1, gastrointestinal (GI) symptoms group; S0, non-gastrointestinal (non-GI) symptoms group*.

We reanalyzed the data, adding HRSD-17 scores as a covariate in the between-group comparisons and we obtained similar results (Supplementary Table 1, Supplementary Figure 1), indicating that the depressive severity had limited effects on the present results.

#### MDD With GI Symptoms vs. HCs

**Figure 4:**
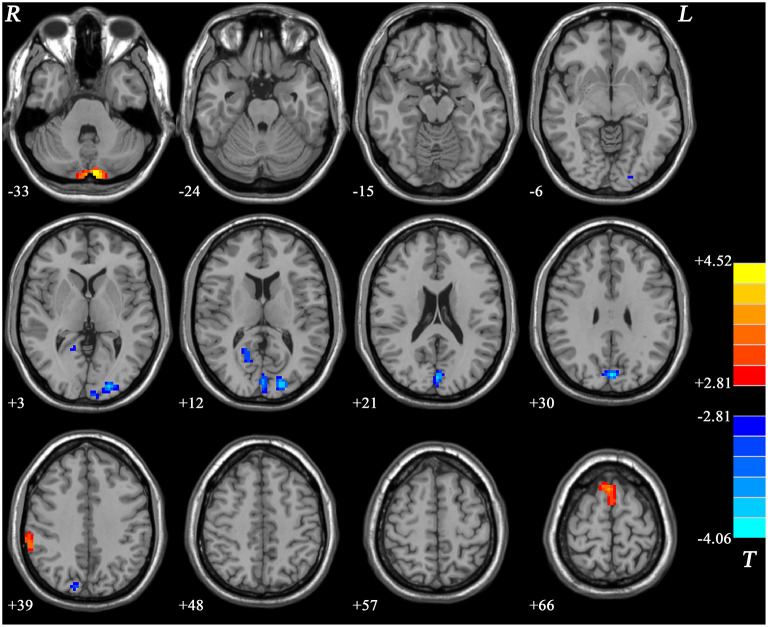
Statistical map depicts higher and lower ReHo in GI group compared with healthy controls. The threshold was FDR (false discovery rate) corrected at *p* < 0.05. Blue denotes lower ReHo and red denotes higher ReHo in the GI group. The color bar indicates *T* values from *post-hoc t*-tests. L, lest side; R, right side; GI, gastrointestinal; ReHo, regional homogeneity.

Compared with the HCs, the GI group showed increased ReHo values in the right inferior parietal lobule (IPL), bilateral supplementary motor area (SMA), and the bilateral cerebellum Crus II and decreased ReHo in the right posterior cingulate cortex (PCC), bilateral cuneus, and the left middle occipital gyrus (MOG) (Figure 4, Table 2).

#### MDD Without GI Symptoms vs. HCs

**Figure 5:**
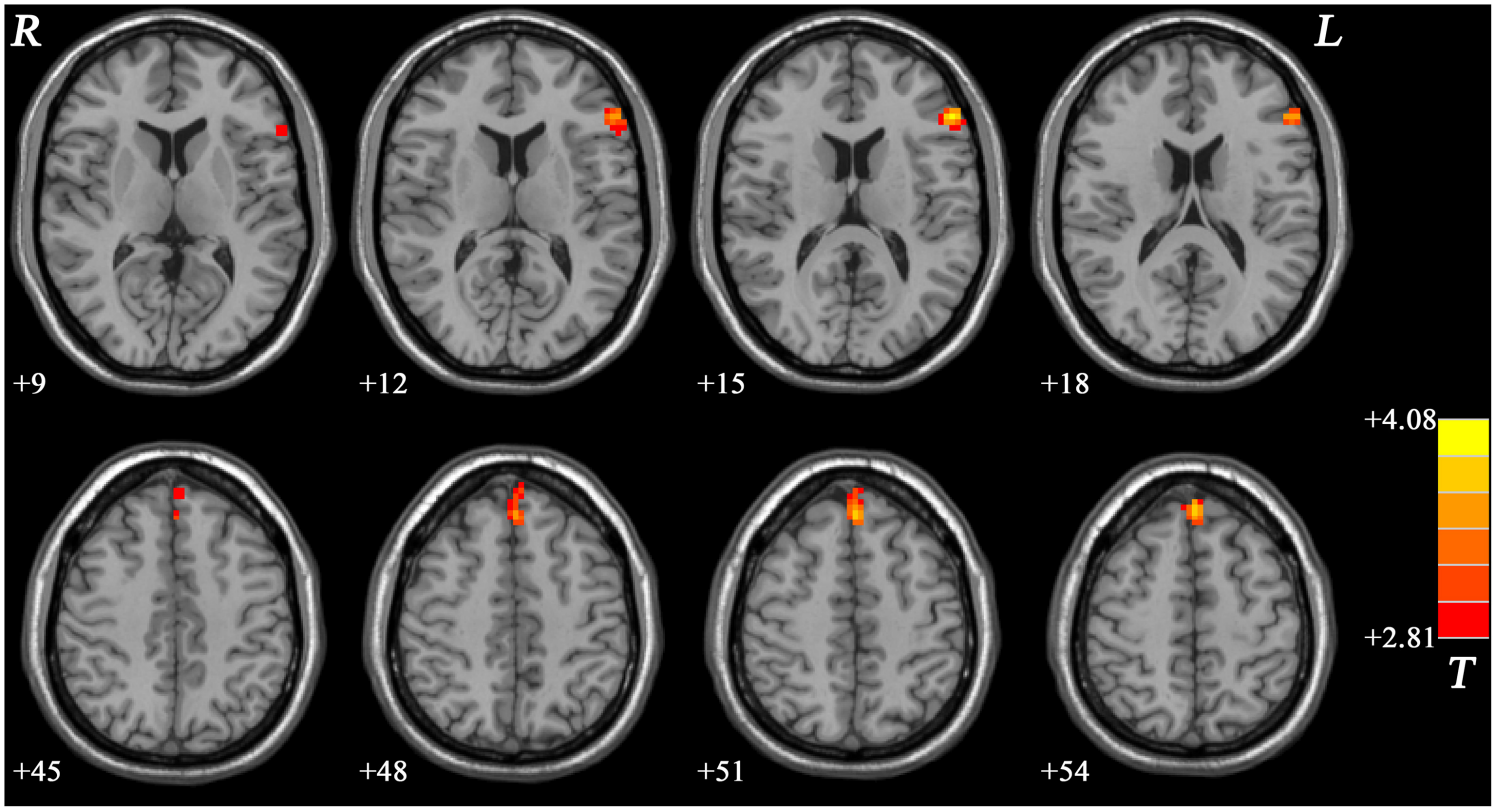
Statistical map depicts higher ReHo in non-GI group compared with healthy controls. The threshold was FDR (false discovery rate) corrected at *p* < 0.05. Red denotes higher ReHo in the non-GI group. The color bar indicates *T* values from *post-hoc t*-tests. L, lest side; R, right side; GI, gastrointestinal; ReHo, regional homogeneity.

Compared with HCs, the non-GI group showed increased ReHo in the left inferior frontal gyrus (IFG) and the bilateral superior medial frontal cortex (SMFC) (Figure 5, Table 2). No decreased ReHo in any brain region was found in the non-GI group relative to the HCs.

### Correlations Between ReHo and Clinical Characteristics

For all patients, no correlation was observed between abnormal ReHo values and HRSD-17 total scores or its subscale scores.

For the GI group, decreased ReHo of the left MOG was positively correlated to weight loss scores (*r* = 0.553, *p* = 0.001, Benjamini-Hochberg correction *p* = 0.007) and increased ReHo of the left precuneus was positively correlated to sleep disturbance scores (*r* = 0.488. *p* = 0.003, Benjamini-Hochberg correction *p* = 0.021). No significant correlation was found between abnormal ReHo values and GI symptoms.

For the non-GI group, there was no correlation between abnormal ReHo values and HRSD-17 total scores or its subscale scores.

## Discussion

In this work, we found that the severity of symptoms was greater in MDD patients with GI symptoms than in MDD patients without GI symptoms, particularly in terms of anxiety/somatization, weight loss, and sleep disturbances. Significantly different ReHo values across the three groups were mainly exhibited in the DMN and cerebellar areas. Moreover, we observed that MDD patients with GI symptoms exhibited increased ReHo in the bilateral precuneus compared with MDD patients without GI symptoms. For the GI group, some altered ReHo were correlated with the factor scores of HRSD-17, whereas no significant correlation was found between abnormal ReHo and GI symptoms.

Consistent with previous studies, somatic symptoms were associated with more severe clinical symptoms in patients with MDD (7). In the present study, the GI group showed higher HRSD-17 total scores, anxiety/somatization, weight loss, and sleep disturbance scores than those in the non-GI group, indicating that the MDD patients with GI symptoms showed greater severity of depression than the MDD patients without GI symptoms. A previous study reported that the somatic symptoms could predict a worse prognosis in patients with MDD (8). Thus, early identification of patients with GI symptoms as their chief complaint (i.e., to identify depressive symptoms in those with GI symptoms) and the active management of their depressive symptoms and GI symptoms may have a positive impact on the prognosis of patients and may help reduce the recurrence rate (7).

Lesions of the parietal lobule are associated with memory deficits, in which the right IPL lesions will cause visuospatial short-term memory impairments (45). As one of the most common cognitive disturbances, memory impairment—especially episodic autobiographical memory (46, 47)—is often observed in MDD. Previous studies have indicated that the IPL was involved in episodic autobiographical memory and was active during episodic retrieval (48). Our previous studies reported that drug-naive MDD patients exhibited lower long-range positive FC strength in the right IPL than the HCs (49) and decreased FC between Crus I and the left IPL (50). Patients with MDD were reported to show increased cerebral blood flow (rCBF) in the bilateral IPL relative to HCs (51). In line with those studies, the present study found increased ReHo in the right IPL in the GI group, which might explain the abovementioned memory deficits in MDD to some extent. The hippocampus also participates in the episodic retrieval and the IPL may act as an “episodic buffer” (52, 53). Decreased negative FC in the right hippocampus to the right IPL was observed in patients with MDD compared with HCs (54). Monkey's IPL was reported to express direct reciprocal projections to the parahippocampal cortex (55, 56) and had direct projections to the hippocampus (57). Thus, the correlations between the IPL and the hippocampus in MDD are interesting and reciprocal in episodic retrieval. Changes in the IPL in digestive system-related diseases have been reported in a few studies. Patients with Crohn's disease showed increased FC between the right middle frontal gyrus and the right IPL compared to HCs (58). Functional dyspepsia (FD) patients with depression and anxiety showed altered glucose metabolism in the cortical-limbic regions (including higher glucose metabolism in the right IPL) compared with FD patients without depression and anxiety (59). The abovementioned studies indicated that the right IPL might be involved in the complex process of the vicious cycle between emotional symptoms and GI symptoms.

The supplementary motor area (SMA) consists of the supplementary motor area proper and pre-supplementary motor area (pre-SMA) in humans, whereas these two regions are two separate areas in monkeys (60–63). As outlined in the abovementioned studies, pre-SMA seems to play a key role in cognitive control functions. Furthermore, SMA has important functional connections with cerebellum and basal ganglia and plays a role in receiving and transmitting information between theses brain regions (64, 65). The cerebellum was involved in the process of emotion and cognition (66–69), in which the posterior lobe was believed to play a critical role in this process (69). A previous study showed that cognitive impairments would occur when lesions of the posterior lobe affected lobules VI and lobules VII (containing Crus I, Crus II and lobule VIIB), and disrupted cerebellar modulation of cognitive loops with cerebral association cortices (68). In the present study, we found increased ReHo in both the bilateral SMA and bilateral cerebellum Crus II in the GI group compared with HCs. So, we suspected that these changes might correlate with cognitive disturbances in patients although there was no significant difference in cognitive function between MDD with and without GI symptoms, as roughly estimated by HRSD-17 in the present study. Many previous studies observed brain imaging changes in the cerebellum in patients with MDD, such as altered gray matter (70, 71), FC, and fALFF (50, 72). Thus, it was not just a coincidence that both SMA and the cerebellum showed an abnormal ReHo at the same time in the MDD patients. Both abnormalities of SMA and the cerebellum may be involved in the pathophysiology of patients with MDD. In addition, increased connectivity was observed in the left SMA in patients with functional gastroenterological diseases compared with HCs, which was interpreted as “the GI symptoms might be reactions to auditory and imaginary stimuli” (31), because the SMA was reported to support a flexible participation in the sensorimotor processes to enable auditory and imaginary perception (73). Conformably, higher ReHo in the bilateral SMA was observed in MDD patients with GI symptoms than that in HCs in the present study. Thus, the SMA may play a role in the mechanism of the GI symptoms in patients with MDD.

PCC locates in the posterior DMN and is involved in processes of memory and problem-solving tasks (74, 75). Previous studies have reported structural and functional brain imaging changes in the PCC. For example, our previous study revealed decreased voxel-mirrored homotopic connectivity (VMHC) in the PCC and cuneus in MDD patients and a combination of VMHC values of these two clusters could separate patients from HCs with good sensitivities and specificities (27, 76). Cuneus is a smaller part of the occipital lobe consolidating information into visual working memory (77). A previous study reported that the occipital bending was more common in MDD patients than that in HCs, and enlargement of the ventricle may aggravate the natural curvature of the occipital regions (78). In another study in melancholic depression, researchers observed asymmetrical enlargement of the CSF space in the Sylvian fissure region in patients (22). Female patients with MDD were reported to show decreased ALFF in the left MOG relative to HCs (79). MDD patients also showed decreased VMHC in the MOG and cuneus (80). In line with these abovementioned results, we observed decreased ReHo in the right PCC, bilateral cuneus, and left MOG in the GI group relative to HCs. Decreased ReHo values of the left MOG in the GI group was also positively correlated with weight loss scores. Although we applied different criteria to recruit different types of MDD patients, we could speculate that an abnormal structural and functional changes in both the PCC and occipital gyrus may be stable features in MDD.

The superior frontal gyrus, an important part of prefrontal gyrus, plays a key role in self-consciousness, emotional regulation, and cognitive processes (81, 82). The medial prefrontal cortex (MPFC) engages in self-referential processing (83). Our previous study observed lower coherence-based ReHo (Cohe-ReHo) in the bilateral frontal gyrus in both treatment-sensitive and treatment-resistant depression (84). Both MDD patients with and without generalized anxiety disorder (GAD) comorbidity presented with cortical thinning in the bilateral middle frontal cortex, left medial frontal gyrus and frontal pole (85). Higher network homogeneity (NH) of the left MPFC was observed in MDD compared to the HCs in a replication study (86). Higher FC in the IFG was observed in medication-free MDD (87). In the present study, we observed increased ReHo in the left IFG and bilateral SMFC in the non-GI group compared with HCs. Although we could not compare our results with previous studies directly, because of different methods and inclusive/exclusion criteria (our subjects were the MDD patients without GI symptoms, instead of all subtypes of MDD), the aforementioned and present results suggest that the frontal gyrus plays an important role in MDD.

As one of the main brain regions of the DMN, the precuneus plays a central role in the neural networks associated with consciousness like self-reflection processes and episodic memory retrieval (88, 89). Abnormal structural and functional changes in the precuneus in patients with MDD were observed in many previous studies. For example, some results showed increased activity like higher FC than HCs (90) and some exhibited decreased activity like decreased ReHo (91), FC (24, 92), and VMHC (27, 93). In the present study, the GI group exhibited higher ReHo values in the bilateral precuneus than the non-GI group, which was inconsistent with the previous study (32) which observed no significant difference in the precuneus between the two patient groups. Different scan parameters and sample heterogeneity (i.e., 35 years old in the previous study and 30 years old in the present study) might lead to the discrepancy. Precuneus would deactivate when sleeping (88), so we suspected that abnormal ReHo in the precuneus may correlate with greater severity of sleep disturbances in the GI group. Indeed, the results of the correlation analyses showed that the increased ReHo of the left precuneus was positively correlated with sleep disturbance scores in the GI group. In Figure 3, a significant difference in the bilateral precuneus was only shown between MDD patients with and without GI symptoms, whereas no significant difference was found between both patient groups and the HCs. We suspected that brain imaging changes of the precuneus are a complex process when emotional symptoms and GI symptoms are present in the same person. A previous study suggested that brain regions involved in pain sensory processing shifted to those involved in subjective states of emotion and motivation in the majority of chronic pain diseases (36). In patients with functional constipation, significantly lower fractional anisotropy (FA) values were observed between the right thalamus and the right precuneus than those in the HCs (94), and a powerful structural relationship was found between the two regions which could act as a modulating pathway during impaired body consciousness (95). Patients with IBS showed apparent differences in brain activation patterns in the precuneus during the rectal vs. heterotopic stimulation comparison (29). The precuneus also showed greater activation associated with unexpected pain intensity (96). The abovementioned studies indicated that the alterations of the bilateral precuneus may be responses of the chronic GI symptoms in patients with MDD only, because the precuneus shows no altered ReHo in patient groups compared to HCs in the present study.

Our study has some limitations. First, the sample was small; second, we did not further classify whether patients with different GI symptoms showed different ReHo. Finally, we could not elucidate whether the changes in ReHo were prior to or as a result of gastrointestinal symptoms. If it were a pre-existing abnormality, we could use the neuroimaging marker to identify patients who had gastrointestinal symptoms and we could have provided them with early intervention to improve their prognosis.

## Conclusion

In conclusion, MDD patients with GI symptoms showed a greater severity of symptoms than MDD patients without GI symptoms, particularly in terms of anxiety/somatization, weight loss, and sleep disturbances. Increased activity in the DMN might be associated with GI symptoms in MDD patients.

## Data Availability Statement

The raw data supporting the conclusions of this article will be made available by the corresponding authors, without undue reservation.

## Ethics Statement

The studies involving human participants were reviewed and approved by the Medical Research Ethics Committee of the Second Xiangya Hospital of Central South University, China. The patients/participants provided their written informed consent to participate in this study.

## Author Contributions

All authors contributed and approved the final manuscript.

## Conflict of Interest

The authors declare that the research was conducted in the absence of any commercial or financial relationships that could be construed as a potential conflict of interest.
