# Supplementary material for: Disrupted Regional Homogeneity in Major Depressive Disorder With Gastrointestinal Symptoms at Rest
Source: Front Psychiatry. 2021 May 26;12:636820. doi: 10.3389/fpsyt.2021.636820 (PMC8187583; doi:10.3389/fpsyt.2021.636820)
Supplement: Supplementary file 1 [file Data_Sheet_1.zip › Table 1.DOCX]

Table S1. Significant ReHo differences between two patient groups (age, years of education, framewise displacement, and HRSD-17 scores as covariates).

| Cluster location | Peak (MNI) | | | Number of voxels | *T* value |
| --- | --- | --- | --- | --- | --- |
|  | x | y | z |  |  |
| *S1 vs S0 (Figure S1)* |  |  |  |  |  |
| Left Precuneus | -15 | -54 | 36 | 30 | 3.6055 |
| Right Precuneus | 12 | -54 | 36 | 48 | 4.0058 |

MNI = Montreal Neurological Institute; ReHo = regional homogeneity.


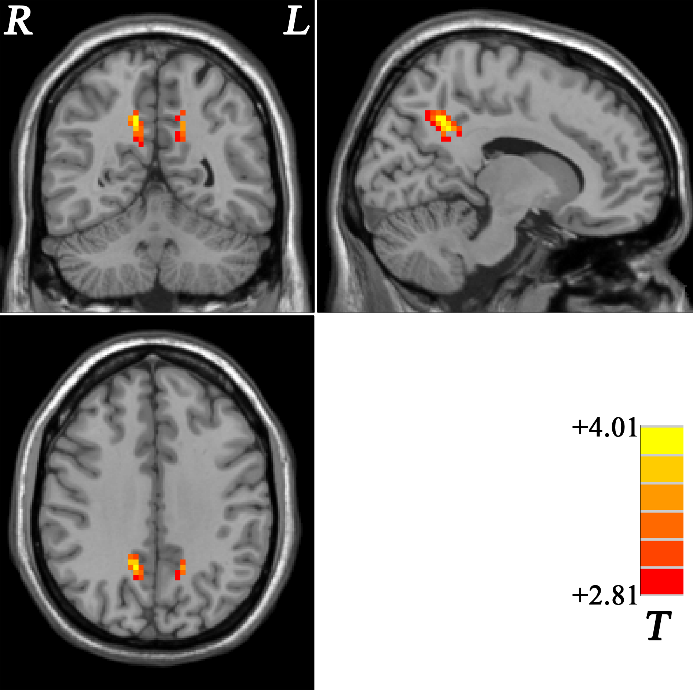


Figure S1. Statistical map depicts higher ReHo in GI group compared with non-GI group (age, years of education, framewise displacement, and HRSD-17 scores as covariates). The threshold was FDR (false discovery rate) corrected at p <0.05. Red denotes higher ReHo in the GI group. Color bar indicates T values from post-hoc t-tests. L=lest side; R=right side; GI =gastrointestinal, ReHo=regional homogeneity.
